# The SNARC effect is associated with worse mathematical intelligence and poorer time estimation

**DOI:** 10.1098/rsos.172362

**Published:** 2018-08-08

**Authors:** Peter Kramer, Paola Bressan, Massimo Grassi

**Affiliations:** Dipartimento di Psicologia Generale, Università di Padova, Via Venezia 8, 35131 Padova, Italy

**Keywords:** SNARC, absolute SNARC, time estimation, mathematical skill, mental rotation

## Abstract

Interactions between the ways we process space, numbers and time may arise from shared and innate generic magnitude representations. Alternatively or concurrently, such interactions could be due to the use of physical magnitudes, like spatial extent, as metaphors for more abstract ones, like number and duration. That numbers might be spatially represented along a mental number line is suggested by the SNARC effect: faster left-side responses to small single digits, like 1 or 2, and faster right-side responses to large ones, like 8 or 9. Previously, we found that time estimation predicts mathematical intelligence and speculated that it may predict spatial ability too. Here, addressing this issue, we test—on a relatively large sample of adults and entirely within subjects—the relationships between (a) time: proficiency at producing and evaluating durations shorter than one second, (b) space: the ability to mentally rotate objects, (c) numbers: mathematical reasoning skills, and (d) space–number associations: the SNARC effect. Better time estimation was linked to greater mathematical intelligence and better spatial skills. Strikingly, however, stronger associations between space and numbers predicted worse mathematical intelligence and poorer time estimation.

## 1. Introduction

With the increasing importance of technology in our society comes an increasing demand for mathematical skills—and, in the hope of improving these, the need to better understand their psychological bases. We have previously shown that the ability to estimate time durations predicts mathematical intelligence (the ability to solve mathematical problems under time pressure) but not non-mathematical, conceptual intelligence (the ability to judge under time pressure what different items, such as air and water, have in common) [1]. We suggested that the relationship between time estimation and mathematical reasoning may be due to a common reliance on spatial representation. Here, after briefly reviewing the main behavioural and neuropsychological findings regarding the interactions between numerical, spatial and temporal processing (for extensive reviews, see [2,3]), we test this hypothesis.

Many researchers have investigated how people mentally represent numbers. The main impetus behind this effort was the discovery, in parity (odd versus even) judgements, of the spatial–numerical associations of response codes (SNARC) effect [4]: faster left-side responses to small single digits, like 1 or 2, and faster right-side responses to large ones, like 8 or 9. (For a meta-analysis across 106 experiments, see [5].) It has been argued that the SNARC effect is due to the reliance on the spatial metaphor of a line, a *mental number line*, on which—in Western societies—numbers increase from left to right because people read and write from left to right. In the majority of people in such societies the SNARC effect is indeed consistent with a left-to-right mental number line—although, curiously, in a sizeable minority it appears consistent with a right-to-left one instead [6].

SNARC-like effects have been found not only in the numerical but also in the temporal domain, or across these two domains, which has been interpreted as reflecting either a mental *time* line or a shared, general mental representation of magnitudes. For example, judgements of whether, after seven clicks separated by the same time interval, an eighth occurs after a shorter or longer interval reveals a *spatial–temporal* association of response codes (STEARC) effect: faster left-side responses to shorter intervals and faster right-side responses to longer ones [7,8]. Likewise, parity judgements of single digits, performed with Morse-code-like short and long responses, show a *temporal–numerical* association of response codes (TiNARC) effect: faster short responses to small single digits and faster long responses to large ones [8,9].

Consistent with these behavioural findings on numerical, spatial and temporal interactions are remarkable neuropsychological ones. For example, patients who, due to right-hemisphere damage, neglect attending stimuli's left side tend to show numerical biases that parallel spatial ones. When asked to bisect a line (i.e. to divide it exactly in the middle), they often respond off to the right [10]; when asked to bisect a numerical interval, say from ‘2’ to ‘6’, they typically respond ‘5’—a number that, on a mental number line, is also off to the right ([2,11]; cf. [12]). Neglect of stimuli's left side can be mimicked in healthy participants by letting them adapt to wearing prismatic lenses that shift one's visual field to the right. In this case, when asked immediately afterwards to reproduce a duration, or to bisect it (reproduce half of it), they generate a duration that is too long—and is therefore, on a mental time line, off to the right. Conversely, adapting to wearing prismatic lenses that shift one's visual field to the left results in an estimated duration that is too short— and is therefore, on a mental time line, off to the left ([13,14]; for related work on neglect patients, see [15,16]).

Interactions between space, time and number processing could be due to shared generic magnitude representations—as proposed in A Theory of Magnitude (ATOM: [17,18]). That such representations may be innate is suggested by the fact that time--number interactions have been found in newborns ([19]; see also [20]) and in rats [21]. Alternatively or in addition, these interactions might stem from our understanding of abstract magnitudes, like number and duration, in terms of concrete, physical ones like spatial extent—as proposed in the Conceptual Metaphor Theory [3]. In a similar vein, some have argued that the SNARC effect and analogous phenomena are due to representations of magnitude that associate numerical magnitude not to continuous *visual* space but to categorical or ordinal *verbal* space. In this verbal space, for example, ‘small’ is associated with ‘left’ and ‘large’ with ‘right’ [22]; or, relative to other numbers, a target number is associated with a particular position in the way a letter is with the rest of the alphabet (e.g. [23,24]; cf. [25–27]).

The study we report here is an extension of Kramer *et al.* [1] and for this reason we measure mathematical and non-mathematical intelligence in the same way as we did before. Time estimation is measured in two ways. In what we call *time evaluation* (the same measure we used previously), participants judge the duration in milliseconds of a given tone duration. In what we call *time production*, participants produce the tone duration corresponding to a given number of milliseconds. We now also measure spatial ability (as the ability to mentally rotate depicted objects) and the SNARC effect, which is thought to reflect the strength of people's associations between space and numbers.

As mentioned, a substantial minority of those who read and write from left to right reveals, nonetheless, a SNARC effect that is consistent with a right-to-left mental number line. This reversed SNARC is traditionally treated as an anti-SNARC; that is, as farther away from the regular SNARC than a SNARC effect of zero. We believe this is a mistake. It leads to paradoxes, such as that a population where half the people displayed a regular SNARC effect, and the other half an equally strong reversed one, would be considered to show no SNARC effect on average. Unlike the absence of a SNARC effect, the regular and reversed SNARC effects are both consistent with the participant being influenced, during parity judgement, by irrelevant numerical magnitudes. If one accepts the hypothesis that numerical magnitude is represented along a mental number line, the only difference between a regular and a reversed SNARC is the direction of this line. Therefore, although we do analyse the SNARC effect, here we introduce and pay special attention to a version of it—the |SNARC| effect—that disregards the direction of the (presumed) underlying mental number line.

## 2. Methods

### 2.1. Participants

Participants were 151 adults (77 women and 74 men; mean age 23.7, median age 23.0, range 18–60 years), mostly university students of a diverse range of disciplines.

### 2.2. Apparatus and materials

The time evaluation and time production experiments were custom-programmed using Matlab's Psychophysics Toolbox [28] and PSYCHOACOUSTICS extension [29]. The SNARC experiment was custom-programmed using E-Prime 2.0 software. All three experiments were run on an ASUS Pentium IV computer connected to a NEC MultiSync FE950+ monitor and an M-AUDIO FastTrack Pro sound card. The output of the sound card was delivered to a pair of Sennheiser HD 580 headphones, at 65 dBA measured at the subject's ear. As in our earlier study [1], all tones presented during the experiments had a 16-bit resolution, a sample rate of 44.1 kHz, and were amplitude-steady ones, gated on and off with 10-ms raised cosine ramps (to avoid onset and offset clicks). The tone included the first four harmonics of a 250-Hz fundamental frequency. The participant was seated at a distance of 60 cm from the monitor, at a small table, inside a single-walled IAC soundproof booth.

Mathematical and non-mathematical (i.e. conceptual) intelligence were respectively assessed with the arithmetic and similarities subscales of the Wechsler Adult Intelligence Scale Revised (WAIS-R); and visual spatial ability with Vandenberg and Kuse's [30] mental rotation test, which uses Shepard and Metzler's [31] stimuli.

### 2.3. Procedure

Participants completed, in order, the two time estimation tasks (time evaluation and time production), the parity judgement task, the two WAIS-R tasks (WAIS-R arithmetic and WAIS-R similarities) and the mental rotation task.

#### 2.3.1. Time estimation

Participants were first reminded that a millisecond is a thousandth of a second and then performed the time evaluation and production tasks. Presented durations were all below 1 s and this prevented the duration-estimation strategy of counting seconds, while also minimizing working memory load. Following Kramer *et al.* [1], participants were not provided with feedback, training sessions or anchors.

In each trial of the time evaluation task, participants heard one of four tones that were identical except for their duration (100, 200, 400 and 800 ms). After each tone, they typed their estimate of its duration in milliseconds. Each tone was repeated six times for a total of 24 trials, randomly intermixed.

In each trial of the time production task and for the whole duration of the trial, participants saw at the centre of the screen one of four numbers (100, 200, 400 and 800) representing a target duration in milliseconds. The number was accompanied by a tone whose duration participants were asked to adjust to match the written one. The tone's initial duration was set randomly between 20 and 1600 ms (the minimum and maximum value to which the participants could adjust it). Participants clicked on either the upper or lower half of the screen to increase or decrease, respectively, the tone's duration. The further above or below the screen's centre participants clicked, the greater the increase or decrease. After each click, the tone was presented with its new duration and participants could click again to make a further adjustment or, if satisfied, click on the extreme right of the screen to save the trial and move on to the next one. Each written target duration was repeated five times for a total of 20 trials, randomly intermixed.

#### 2.3.2. Parity judgement

Each trial started with the presentation of a central fixation point that, after 500 ms, was replaced by a digit (1, 2, 3, 4, 6, 7, 8 or 9). Half the participants were to press a left key (z) if the digit was odd and a right key (m) if it was even; half were to do the converse. Participants were asked to respond as fast as possible but avoid errors. The experiment started after a training session of 16 trials in which feedback was provided. Each digit was presented 20 times, in random order, for a total of 160 trials. The trials were divided by a self-paced break into two blocks of 80.

#### 2.3.3. WAIS-R

As in our previous work [1], mathematical and non-mathematical (i.e. conceptual) intelligence were measured with the Italian version of the arithmetic and similarities subtests of the WAIS-R. The arithmetic subtest involves solving, under time pressure, arithmetic problems from easy (e.g. ‘What is the total of 4 plus 5 apples?’) to relatively hard (e.g. ‘If 8 machines can finish a job in 6 days, how many machines are needed to finish it in half a day?’). The final score can range from 0 to 20. The similarities subtest requires solving, also under time pressure, non-mathematical problems from easy (‘In what way are an orange and a banana alike?’) to relatively hard (‘In what way are praise and punishment alike?’). The final score can range from 0 to 28.

#### 2.3.4. Mental rotation

Participants viewed a series of 20 target images of three-dimensional objects [30]. Each of them was accompanied by four images: two were rotated images of the target and two were rotated images of one or two of the other 19 targets. The participant was asked to indicate which two of these four images matched the target. Failure to correctly identify one of them, or both, counted as an error.

### 2.4. Data analysis

The study was conducted by four experimenters, each with a different, experimenter-recruited, subset of participants and at different points of time over a period of 5 years. Indeed, mean errors in time production and especially evaluation turned out to differ between experimenters, possibly due to small variations in delivering the instructions for these tasks.^1^ To eliminate any experimenter effect, we transformed final evaluation and production errors (along with all our other variables: SNARC, |SNARC|, WAIS-R scores and mental rotation score) into *z*-scores, separately for each experimenter, and used these standardized measures in all statistical analyses.

Data were analysed using linear regressions and *t*-tests. Because some of the independent variables in the regressions were correlated, we checked for potential collinearity issues but found none. All quoted tests are two-tailed.

#### 2.4.1. Time estimation

One participant misunderstood the time evaluation task and the corresponding data were therefore excluded. For each trial, we calculated the absolute value of the time evaluation error (difference between estimated and physical duration divided by the physical duration) and the absolute value of the time production error (difference between produced and physical duration divided by the physical duration). For each participant and tone duration, we averaged across the six time evaluation errors and, separately, across the five time production errors. In the time evaluation condition, four data points (out of 3600) appeared to be typos and were excluded from the means (including them had virtually no effect on the results). For each participant and duration, we thus obtained one final time evaluation error and one final time production error, which were then *z*-transformed as described above. The standardized evaluation and production errors were also averaged to obtain a single, more general, time estimation error per participant and duration.

#### 2.4.2. SNARC and |SNARC|

Owing to technical error, one participant's parity judgement data were lost. Another participant's responses in the parity judgement experiment suggested that she had inverted her response assignment. We treated her data as if this were indeed the case. Inclusion or exclusion of the data of this participant had virtually no effect on the results.

In the parity judgement experiment, the SNARC effect consists of faster left-side responses to small numbers and faster right-side responses to large ones. If response assignment is varied not within subjects [32] but between them, like in our current study, one way to analyse the data is to bin responses to pairs of numbers. A previous study [33] used the numbers 2 to 9, rather than 1 to 9 excluding 5, and binned the pairs 2 and 3, 4 and 5, 6 and 7, and 8 and 9. For half of the participants, the smaller number within each bin required a left-key response and the larger one a right-key response. For the other half, the converse was true. For each participant and bin, the difference in reaction time was then calculated between the mean right-key and mean left-key reaction times: dRT = RT right key – RT left key. Next, the dRT values were regressed on magnitude bin, with negative regression slopes consistent with a left-to-right mental number line.

This calculation method has the disadvantage that magnitude differences within, rather than between, bins are ignored. That is, the method treats the reaction time differences between right- or left-key responses to the smaller number within each bin, and respectively, left- or right-key responses to the larger one, as if they were reaction time differences between responses to identical numbers. Yet, the smaller and the larger number within each bin are not identical, and—on the basis of the SNARC effect—one would predict right-key responses to be slightly slower, and left-key responses to be slightly faster, to the smaller than to the larger number. Not taking this systematic variance into account may introduce noise.

Here, we avoided binning. For each participant and digit in the parity judgement experiment, we calculated the median response time (incorrect responses and responses provided after 1500 ms were excluded). For those who responded ‘odd’ with the left key and ‘even’ with the right one, a SNARC effect is then reflected by a median response time that increases with the magnitude of odd numbers and decreases with that of even ones. For each participant, we therefore calculated the slope of the linear regression between odd numbers and their associated median response times, which should be positive, and between even numbers and theirs, which should be negative, multiplied the latter slope by −1, and then averaged both slopes to obtain a single SNARC effect. We performed the symmetrical calculation for those who responded ‘even’ with the left key and ‘odd’ with the right one. Note that, with our procedure, positive SNARC effects are consistent with a left-to-right mental number line and negative SNARC effects with a right-to-left one. This choice leads, we feel, to a more intuitive measure in which a greater effect is represented by a larger positive value.

If the two regression slopes of a participant are both positive (one straight away and one after multiplication by −1), their average—the participant's SNARC effect—is also positive (consistent with a left-to-right mental number line). Each regression slope measures how much a participant is influenced, during parity judgement, by irrelevant numerical magnitudes. If, however, one slope (or both) is negative rather than positive, such a slope still measures this influence, even though the SNARC effect may now be very small, zero or even negative (consistent with a right-to-left mental number line). To allow a test of whether time evaluation and production are affected by irrelevant numerical magnitudes in whichever direction, rather than only left-to-right as in the SNARC effect, we computed the average of the *absolute* values of the two slopes, the |SNARC| effect.

## 3. Results

### 3.1. Descriptives

Descriptive statistics are presented in table 1. Note that the |SNARC| and SNARC effects were both significantly larger than zero, respectively *t*_149_ = 18.43 and *t*_149_ = 12.46, both *p* < 0.0001. Of the participants, 85% had a (positive) SNARC effect consistent with a left-to-right mental number line, and 15% a (negative) SNARC effect consistent with a right-to-left mental number line.^2^

**Table 1.** Descriptive statistics. Positive values for the SNARC effect are consistent with a left-to-right mental number line.

task	mean	standard deviation	minimum	maximum
|SNARC|	7.64	5.08	0.38	30.15
SNARC	6.07	5.97	−8.15	23.06
WAIS-R arithmetic	11.58	2.72	5	19
WAIS-R similarities	19.70	3.47	8	28
mental rotation	9.44	4.63	0	20
time estimation error (%)	53	34	14	186

### 3.2. Time

#### 3.2.1. Time estimation

**Figure 1.**
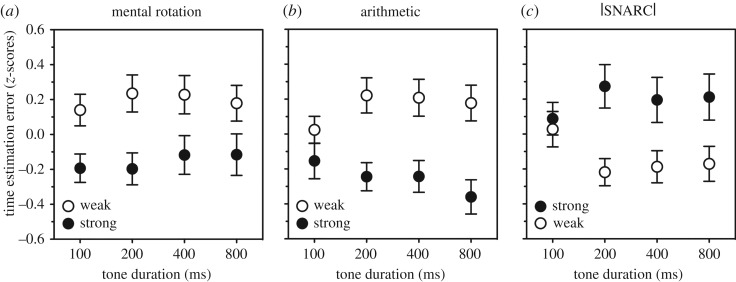
Time estimation error for each of the four tone durations used in the experiments, separately for participants with either a strong (highest tertile, closed symbols) or a weak (lowest tertile, open symbols) mental rotation ability (*a*), mathematical intelligence (*b*) and |SNARC| effect (*c*). Tertiles are shown for illustrative purposes but were not used in the analyses.

The combination of |SNARC|, mental rotation ability and mathematical intelligence (WAIS-R arithmetic score) predicted 19% of the variance in time estimation error (*R* = 0.44, *F*_3,146_ = 11.72, *p* < 0.0001), with all three variables contributing to the overall effect (|SNARC|: *β* = 0.20, *t* = 2.58, *p* = 0.011; mental rotation ability: *β* = −0.23, *t* = −2.91, *p* = 0.004; mathematical intelligence: *β* = −0.21, *t* = −2.59, *p* = 0.011). Note that whereas mental rotation ability and mathematical intelligence both decreased time estimation error, |SNARC| increased it. Figure 1 shows that results were quite similar for each of the tone durations separately. Adding conceptual intelligence (WAIS-R similarities score) to the regression model hardly changed any of these results—and the variable did not reach significance. In all analyses, replacing |SNARC| with SNARC led to weaker but otherwise similar results. For a correlation matrix of all these variables, see table 2.

**Table 2.** Pearson correlation matrix (after standardization of all measures into *z*-scores) of participants' absolute SNARC effect, SNARC effect, WAIS-R arithmetic score, WAIS-R similarities score, mental rotation score and time estimation absolute error.

task	|SNARC|	SNARC	WAIS-R arithmetic	WAIS-R similarities	mental rotation	time estimation error
|SNARC|		0.76**	−0.23**	−0.19*	−0.07	0.26**
SNARC	0.76**		−0.17*	0.02	−0.02	0.19*
WAIS-R arithmetic	−0.23**	−0.17*		0.09	0.35**	−0.34**
WAIS-R similarities	−0.19*	0.02	0.09		0.15	−0.09
mental rotation	−0.07	−0.02	0.35**	0.15		−0.32**
time estimation error	0.26**	0.19*	−0.34**	−0.09	−0.32**	

*Correlation is significant at the 0.05 level (two-tailed);

**Correlation is significant at the 0.01 level (two-tailed).

#### 3.2.2. Time production

The combination of |SNARC|, mental rotation ability and mathematical intelligence predicted 15% of the variance in *time production* error (*R* = 0.39, *F*_3,146_ = 8.75, *p* < 0.0001), with all three variables contributing to the overall effect (|SNARC|: *β* = 0.19, *t* = 2.48, *p* = 0.014; mental rotation ability: *β* = −0.17, *t* = −2.12, *p* = 0.036; mathematical intelligence: *β* = −0.20, *t* = −2.39, *p* = 0.018). Adding conceptual intelligence to the regression model hardly changed any of these results—and the variable did not reach significance. In all analyses, replacing |SNARC| with SNARC led to weaker but otherwise similar results.

#### 3.2.3. Time evaluation

The combination of |SNARC|, mental rotation ability and mathematical intelligence predicted 10% of the variance in *time evaluation* error (*R* = 0.31, *F*_3,145_ = 5.23, *p* = 0.002), but only mental rotation ability (*β* = −0.19, *t* = −2.31, *p* = 0.022) contributed significantly to the overall effect, whereas neither |SNARC| (*β* = 0.12, *t* = 1.49, *p* = 0.137) nor mathematical intelligence (*β* = −0.13, *t* = −1.53, *p* = 0.128) did. By themselves, mental rotation ability, mathematical intelligence and |SNARC| predicted, respectively, 6% (*R* = 0.25, *p* = 0.002), 6% (*R* = 0.24, *p* = 0.003) and 3% (*R* = 0.17, *p* = 0.042) of the variance. Neither conceptual intelligence by itself nor SNARC by itself predicted any.

### 3.3. Space and number

The combination of time estimation, mental rotation ability, mathematical intelligence and age (age played no role in the previous regressions) predicted 15% of the variance in |SNARC| (*R* = 0.39, *F*_4,145_ = 6.32, *p* = 0.0001). Only time estimation error (*β* = 0.24, *t* = 2.83, *p* = 0.005), mathematical intelligence (*β *= −0.22, *t* = −2.58, *p* = 0.011) and age (*β* = 0.24, *t* = 3.07, *p* = 0.003) contributed significantly to the overall effect, whereas mental rotation ability did not (*β* = 0.10, *t* = 1.15, *p* = 0.251). Without mental rotation ability, the combination of time estimation, mathematical intelligence and age predicted 14% of the variance (*R* = 0.38, *F*_3,146_ = 7.97, *p* < 0.0001), with significant contributions from time estimation error (*β* = 0.21, *t* = 2.63, *p* = 0.009), mathematical intelligence (*β *= −0.19, *t* = −2.35, *p* = 0.020) and age (*β* = 0.23, *t* = 2.94, *p* = 0.004). Adding conceptual intelligence to either of the two regression models hardly changed any of their results, although the variable did reach marginal significance in both. Replacing |SNARC| with SNARC led to weaker but otherwise similar results if mental rotation ability was retained in the regression model; if it was not, the statistical significance of both time estimation and mathematical intelligence became marginal. (For similar findings of a positive relationship between age and the SNARC effect and explanations of it, see [36,37].)

## 4. Discussion

Replicating our previous results [1], we found that time estimation was linked to mathematical intelligence. Consistent with our earlier hypothesis, time estimation turned out to be linked also to spatial ability (here, the ability to mentally rotate depicted objects). These results are in tune with the recent findings that, in children, time *discrimination* correlates with both arithmetic skill and competence at doing mental rotation [38,39], and time *reproduction* correlates with both arithmetic skill and the ability to remember the spatial locations of briefly presented dots [40]. In children, mathematical deficits are also associated with poorer time estimation (e.g. [41,42]).

Our time production results were stronger than our time evaluation ones. A possible reason for this discrepancy might be that participants' time evaluations, unlike productions, tended overwhelmingly to be multiples of 100 ms—which diluted between-subject differences and made the evaluation task less discriminative. Indeed, of a total of 3596 time evaluations, 93% were multiples of 100 ms, whereas of a total of 3019 time productions, only 5% were. The two conditions also differed in task demands. In the time production task, participants were asked to produce a sound of a duration that, in the form of a number, remained visible on the screen till the end of the trial. In the time evaluation task, instead, participants were asked to produce a number representing the duration of a sound that had been heard already and was being kept in memory. This involvement of memory might have been a potential source of noise in performance.

Our data speak not only to the issue of time estimation but also to that of spatial--numerical associations. First, in our sample of 150 participants, we found that the strength of such associations is unrelated to spatial skill, as assessed by proficiency at mentally rotating objects in three-dimensional space. Our result supports a previous study that found, in a sample of 36 participants, an association between the SNARC effect and mental rotation ability in two dimensions, but—like us—not in three dimensions [34].

Secondly we found, in our relatively large sample, that the |SNARC| effect is associated with worse mathematical intelligence. There has previously been no consensus among studies related to ours (see [35]). Of the three most recent ones, the first reported no relationship between the SNARC effect and being proficient at verifying equations in 71 participants [35]. The other two found, respectively, a negative relationship between SNARC and calculation abilities in 95 university students [43] and no significant SNARC effect in 14 professional mathematicians [44]. Unlike ours, all previous studies treated positive and negative SNARC effects as though they were opposites. Here we found, however, that *all* associations of the SNARC effect with other variables were larger when the absolute value of the effect was taken; that is, when positive and negative SNARC effects were treated as equivalent rather than as opposites. In fact, we see no reason why a right-to-left SNARC effect should be seen as anything less of a spatial--numerical association than a left-to-right one.

It appears that no one, before us, has studied the relationship between the |SNARC| (or SNARC) effect and time estimation. It is remarkable that our findings suggest that this relationship mirrors the one between the |SNARC| effect and mathematical intelligence.

Despite correlating significantly with the |SNARC| effect on its own, conceptual intelligence was an only marginally significant predictor of it in our multiple regressions. Our results are thus inconclusive about this association. On the other hand, mathematical intelligence and time estimation predicted the |SNARC| effect both on their own and in multiple regressions—and neither variable was at all related to conceptual intelligence. These results, taken together, exclude that the associations we found are simply due to a better performance of more intelligent people across tasks.

We conclude that the ability to estimate time is better in people who are good at manipulating numbers and representing objects in space, but worse in people for whom associations between numbers and space are stronger. Likely for the same reason, people with a stronger |SNARC| effect turn out to have a weaker mathematical intelligence. If children are young enough, encouraging them to think of numbers as locations on a physical line improves their quantitative skills (e.g. [45,46]). If children are young enough, their SNARC effect is indeed positively related to such skills [47]. The findings we have presented here, however, suggest that, in adulthood, continuing to picture numbers in such a unidimensional way could be not only useless [48] but even counterproductive.

## Footnotes

1 There were no significant differences between the subsets of participants in any of the other tasks (SNARC, mental rotation, WAIS-R arithmetic, WAIS-R similarities). The problematic task was time evaluation, where the errors of one experimenter's participants had a much higher mean and standard deviation than those of the others. Using the untransformed data while excluding the 24 participants run by this experimenter (*N *= 126) led to the same pattern of results as using the *z*-transformed data from all experimenters (*N *= 150). Here, we conservatively present the latter analyses. (Note that although, for consistency, we employed *z*-transformed scores for all measures, using *z*-transformed scores only for the time-tasks measures and not for the others gave similar results.)

2 The odd/even trials split-half reliability of the standardized SNARC effect (*r *= 0.64; |SNARC| effect, *r *= 0.65; Spearman-Brown corrected) was in the same range as in previous reports (e.g. [34,35]). The reliability of all other measures was above 0.80.

## Data accessibility

Data [49] and analysis scripts are publicly available at https://figshare.com/s/c3822f4b205b70e92dc4.
